# Effects of *Nrf2* silencing on oxidative stress‐associated intestinal carcinogenesis in mice

**DOI:** 10.1002/cam4.672

**Published:** 2016-02-21

**Authors:** Yuh Yokoo, Aki Kijima, Yuji Ishii, Shinji Takasu, Takuma Tsuchiya, Takashi Umemura

**Affiliations:** ^1^ Division of PathologyNational Institute of Health Sciences1‐18‐1 Kamiyoga, Setagaya‐kuTokyo158‐8501Japan

**Keywords:** COX2, intestinal tumorigenesis, KBrO_3_, NRF2, oxidative stress

## Abstract

To assess the risk of colorectal cancer in humans with inactivation of NRF2, *Nrf2*‐proficient (*Nrf2* ^+/+^) and ‐deficient (*Nrf2* ^−/−^) mice were exposed to potassium bromate (KBrO_3_) at concentrations of 750 or 1500 ppm for 52 weeks. Neoplastic proliferative lesions were observed in the small intestine and exhibited accumulations of *β*‐catenin and cyclin D1. The lesions had characteristics similar to those in experimental models of human hereditary colorectal cancer. An additional 13‐week study was performed to examine the role of Nrf2 in the effects of oxidative stress. Significant increase in combined incidences of preneoplastic and neoplastic lesions in *Nrf2* ^−*/*−^ mice administered high‐dose KBrO_3_. In the short‐term study, although 8‐hydroxydeoxyguanosine (8‐OHdG) levels in the epithelial DNA of *Nrf2* ^−/−^ mice at the high dose were significantly lower than those of the corresponding *Nrf2* ^*+/+*^ mice, the difference was very small. mRNA levels of Nrf2‐regulated genes were increased in *Nrf2* ^*+/+*^ mice. Overexpression of cyclooxygenase 2 (COX2) and increased numbers of proliferating cell nuclear antigen (PCNA)‐positive cells in the jejunal crypts were observed in *Nrf2* ^−*/*−^ mice administered high‐dose KBrO_3_. Overall, these data suggested that individuals having single‐nucleotide polymorphisms in *NRF2* may have a risk of colorectal cancer to some extent.

## Introduction

Oxidative stress is thought to be one of the causes of colorectal cancers in humans 1, 2. Indeed, some genes responsible for hereditary colorectal cancer function to repair oxidative DNA damage. Bi‐allelic inherited mutations in *MUTYH,* the base excision repair (BER) enzyme eliminating adenine paired to 8‐OHdG have been found in patients with hereditary colorectal cancer. Lesions containing these mutations are referred to as MUTYH‐associated polyposis (MAP) 3. Additionally, germ‐line mutations in genes encoding proteins involved in recognition of mismatch bases, that is, *MSH2*,* MLH1*,* MLH3*,* MSH6*,* PMS1*, and *PMS2*, were found among patients with hereditary nonpolyposis colorectal cancer (HNPCC) 4, 5. Studies have also suggested that *Msh2* may participate in repair of 8‐OHdG 6, 7.

Recently, the relationships between 8‐OHdG formation and these inherited colorectal cancers have been experimentally demonstrated using *Mutyh*‐deficient (*Mutyh* ^−/−^) or *Msh2*‐deficient (*Msh2* ^−/−^) mice treated with KBrO_3_. Although the induced tumors were derived from the small intestine, the incidences and multiplicities of the tumors were dramatically increased compared with those in corresponding wild‐type mice. Moreover, while G:C‐T:A transversion mutations in *Apc* and *Ctnnb1* (*β*‐catenin) genes extracted from the induced tumors and accumulation of *β*‐catenin in nuclei of the tumor cells have been found in *Mutyh* ^−/−^ mice treated with KBrO_3_, the same mutations in *Apc* have also been found in tumors of patients with MAP. Additionally, G:C‐A:T and A:T‐G:C transition mutations and G:C‐C:G and G:C‐T:A transversion mutations at glycogen synthase kinase 3*β* (GSK3*β*)‐mediated phosphorylation sites in *Ctnnb1* were found in the induced‐tumors in *Msh2*‐deficient mice treated with KBrO_3_, consistent with the mutation sites and their spectra found in patient with HNPCC. Therefore, *Mutyh* ^−/−^ and *Msh2* ^−/−^ mice treated with KBrO_3_ are useful experimental models for investigation of human hereditary colorectal cancer, despite the differences in the target organs 8, 9, 10. Likewise, these data indicate that examination of small intestinal tumors using oxidative stress‐related gene‐deficient mice administered KBrO_3_ will provide valuable information concerning the role of the target gene in colorectal tumorigenesis in humans.

Cellular responses to oxidative stress are partly regulated by the redox‐sensitive transcription factor, nuclear factor erythroid 2‐related factor 2 (NRF2). Under normal physiological conditions, NRF2 is anchored in the cytoplasm by kelch‐like ECH‐associated protein 1 (KEAP1), which also mediates proteasomal degradation of NRF2. Oxidative stress causes dissociation of NRF2 from KEAP1 and causes translocation of NRF2 into the nucleus, where it can bind to Antioxidant Response Elements (AREs). Binding to AREs results in transactivation of ARE containing genes encoding anti‐oxidant‐related enzymes, such as NAD(P)H:quinone oxidoreductase 1 (NQO1), heme oxygenase 1 (HO1) and superoxide dismutase (SOD) 11. Thus, the NRF2‐ARE pathway plays a crucial role in protection against oxidative stress at an early stage. In humans, single‐nucleotide polymorphisms (SNPs) in the promoter region of *NRF2* are thought to be associated with the risk of acute lung injury, ulcerative colitis and gastritis, as shown in epidemiological studies 12, 13, 14. Considering the possible contribution of oxidative stress to colorectal cancer in humans, as mentioned above, clarification of the effects of inactivation of NRF2 on lesions is important for assessment of the risk of colorectal cancer in individuals with SNPs in *NRF2*. To this end, long‐term exposure of *Nrf2* ^−*/*−^ mice to KBrO_3_ was performed after *Mutyh* ^−/−^ or *Msh2* ^−/−^ mouse model. In addition, short‐term exposure of *Nrf2* ^−*/*−^ mice to KBrO_3_ was performed to clarify the role of Nrf2 in the effects of oxidative stress on the small intestine.

## Materials and Methods

### Chemicals

KBrO_3_ (purity: 99%) was purchased from Wako Pure Chemical Industries (Osaka, Japan).

### Animals, diet, and housing conditions

The protocol for this study was approved by the Animal Care and Utilization Committee of the National Institute of Health Sciences. *Nrf2*‐deficient mice with an ICR/129SVJ background established by Itoh et al. (1997) 15 were crossed with ICR mice (Japan SLC, Shizuoka, Japan). Homozygous (−/−) and wild‐type (+/+) littermates were then obtained from the F1 generation and genotyped by polymerase chain reaction (PCR) with DNA taken from the tail of each mouse. All mice were housed in polycarbonate cages (5 mice per cage) with hard wood chips for bedding in a conventional animal facility maintained under conditions of controlled temperature (23 ± 2°C), humidity (55% ± 5%), air change (12 times per hour), and lighting (12 h light/dark cycle). Mice were given free access to a CRF‐1 basal diet (Charles River Japan, Kanagawa, Japan) and tap water.

### Experiment 1 (long‐term exposure to KBrO_3_)

#### Animal treatment

Sixty or 77 six‐week‐old female mice of each genotype were divided into 3 groups (20–27 animals/group). KBrO_3_ was dissolved in distilled water at concentrations of 750 or 1500 ppm, and the prepared drinking water was then given to the animals ad libitum for 52 weeks. Control mice were given distilled water. Dose levels were selected based on the small intestinal carcinogenic dose reported in *Mutyh*‐deficient mice 8, 10 and *Msh2*‐deficient mice 9 and a subacute toxicity study of KBrO_3_ 16. Clinical signs and general appearance were observed daily. Body weights and water consumptions were measured every week until week 5 and every 5 weeks thereafter. At necropsy, animals were killed by exsanguination under methoxyflurane anesthesia, and the entire length of the small intestine was harvested and fixed in 10% neutral‐buffered formalin. Fixed small intestines were Swiss‐rolled and embedded in paraffin, and sectioned.

#### Histopathological examination

Tissue sections of the small intestine were stained with hematoxylin and eosin (HE) for histopathological examination. The evaluation of the neoplastic proliferative lesions was performed according to the international classification of rodent tumors 17.

#### Immunohistochemical staining for *β*‐catenin and cyclin D1

After antigen retrieval by autoclaving with sodium citrate buffer, tissue sections were incubated with monoclonal anti‐ *β*‐catenin antibodies (1:100; Abcam, Cambridge, UK) or monoclonal anti‐cyclin D1 antibodies (1:50; Cell Signaling Technology, Inc., Danvers, MA) at 4°C overnight followed by incubation with a high polymer stain (HISTOFINE Simple Stain, Nichirei Bioscience Inc., Tokyo, Japan) at room temperature.

### Experiment 2 (short‐term exposure to KBrO_3_)

#### Animal treatment

Eleven or 15 six‐week‐old female mice of each genotype were divided into 3 groups (3–5 animals/group). KBrO_3_ was dissolved in distilled water and given to the animals ad libitum for 13 weeks at the same dose as in the long‐term study. At necropsy, animals were euthanized by exsanguination under methoxyflurane anesthesia, and the mucosa of the small intestine was scratched, frozen with liquid nitrogen, and stored at −80°C. A part of the collected mucosa was homogenized in ISOGEN (Nippon Gene, Tokyo, Japan) and stored at −80°C until used for isolation of total RNA.

Additionally, 10 six‐week‐old female mice of each genotype were divided into 2 groups (5 animals/group). KBrO_3_ was dissolved in the distilled water at concentrations of 1500 ppm, and the prepared drinking water was given to the animals ad libitum for 13 weeks. Control mice were given distilled water. At necropsy, animals were euthanized by exsanguination under isoflurane anesthesia (Mylan Inc., Tokyo, Japan), and the entire length of the small intestine was harvested and fixed in 10% neutral‐buffered formalin. Fixed small intestines were Swiss‐rolled and embedded in paraffin, sectioned for immunohistochemical examination of PCNA expression. This experiment was performed a second time with an independent set of 10 mice of each genotype.

#### Measurement of nuclear 8‐OHdG

DNA from the small intestinal mucosa was extracted and digested as described previously 18. Briefly, nuclear DNA from the small intestinal mucosa was extracted with a DNA extractor WB kit (Wako Pure Chemical Co.). For further prevention of auto‐oxidation in the cell lysis step, deferoxamine mesylate (Sigma Chemical, St Louis, MO) was added to the lysis buffer. The DNA was digested into deoxynucleotides by treatment with nuclease P1 and alkaline phosphatase using the 8‐OHdG Assay Preparation Reagent set (Wako Pure Chemical Co.). The levels of 8‐OHdG (8‐OHdG/10^5^dG) were measured by high‐performance liquid chromatography with an electrochemical detection system (Coulochem II; ESA, Bedford, MA) as previously reported 19.

#### RNA isolation and quantitative real‐time PCR for mRNA expression

Total RNA was extracted using ISOGEN according to the manufacturer's instructions. cDNA copies of total RNA were obtained using a High‐Capacity cDNA Reverse Transcription kit (Life Technologies, Carlsbad, CA). PCR was performed with primers for mouse *Nqo1* (coding NAD(P)H:quinone oxidoreductase 1), *Hmox1* (coding heme oxygenase 1), *Gstm1* (coding glutathione S‐transferase mu1), *Gclc* (coding glutamate‐cysteine ligase, catalytic subunit), *Il1b* (coding interleukin 1*β*), and *Tnf* (coding tumor necrosis factor). TaqMan^®^ Rodent GAPDH Control Reagents (Product ID: 4308313) were used as an endogenous reference and amplified with an Applied Biosystems 7900HT FAST Real‐Time PCR Systems (Applied Biosystems, foster city, CA) using TaqMan^®^ Fast Universal PCR Master Mix and TaqMan^®^ Gene Expression Assays (Life Technologies). The expression levels of the target gene were calculated by the relative standard curve method and were determined as ratios relative to *Gapdh* expression. Data are presented as fold‐change values of treated samples relative to those of the distilled water‐treated group of *Nrf2*‐wild‐type mice.

#### Protein extraction, sodium dodecyl sulfate polyacrylamide gel electrophoresis (SDS‐PAGE) and western blotting

The small intestinal mucosae were homogenized with a Teflon homogenizer with ice‐cold RIPA lysis buffer (Wako Pure Chemical Co.) containing a mammalian protease inhibitor cocktail. Samples were centrifuged at 15,000*g* for 30 min, and the resulting supernatants were used in experiments. Protein concentrations were determined using Advanced Protein Assays with bovine serum albumin as a standard. Samples were separated by SDS‐PAGE and transferred to 0.45‐*μ*m PVDF membranes (Millipore, Billerica, MA). For detection of target proteins, membranes were incubated with anti‐NQO1 polyclonal antibodies (1:1000; Abcam), anti‐COX2 monoclonal antibodies (1:1000; Abcam), and anti‐GAPDH polyclonal antibodies (1:3000; Santa Cruz Biotechnology, Inc, Dallas, TX) at 4°C overnight. Secondary antibody incubation was performed using horseradish peroxidase‐conjugated secondary anti‐rabbit or anti‐mouse antibodies at room temperature. Protein detection was facilitated by chemiluminescence using ECL Plus (GE Healthcare Japan Ltd., Tokyo, Japan).

#### Immunohistochemical staining for PCNA

Immunohistochemical staining was performed using monoclonal anti‐mouse PCNA antibodies (1:100; Dako Denmark A/S, Glostrup, Denmark) followed by incubation with a high polymer stain (HISTOFINE Simple Stain, Nichirei Bioscience Inc.). Ten crypts from the duodenum, jejunum, and ileum were randomly selected, PCNA‐positive cell numbers per crypt were counted.

### Statistical analysis

Survival rates and incidences of histopathological findings were compared with Fisher's exact probability tests or Chi‐squared tests. Body weights, multiplicities of histopathological findings, 8‐OHdG levels, and mRNA expression levels were analyzed with ANOVA, followed by Dunnett's multiple comparison tests. PCNA‐positive cell numbers per crypt and comparisons between the genotypes for these data were analyzed by Student's *t*‐test or Welch's test depending on the homogeneity. *P* values of less than 0.05 were considered significant.

## Results

### Long‐term exposure to KBrO_3_

#### Survival rates

**Figure 1:**
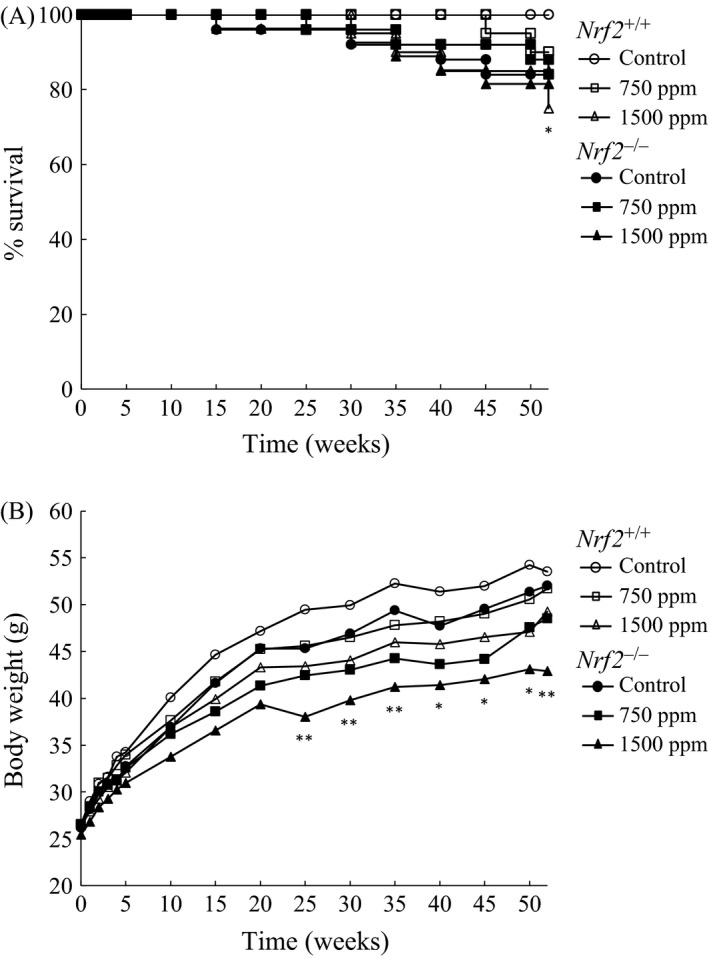
(A) Survival curves for *Nrf2* ^+/+^ and *Nrf2* ^−/−^ mice treated with KBrO_3_. (B) Growth curves for *Nrf2* ^+/+^ and *Nrf2* ^−/−^ mice treated with KBrO_3_. *,**Significantly different from relevant control group at *P *<* *0.05 and 0.01, respectively.

The survival curves for *Nrf2* ^+/+^ and *Nrf2* ^−/−^ mice treated with KBrO_3_ for 52 weeks are illustrated in Figure 1A. In *Nrf2* ^+/+^ mice treated with 1500 ppm KBrO_3_, survival rate of this group dropped to 75% on week 52, being significantly different from the relative control group. There were no significant changes in survival rates in *Nrf2* ^−/−^ mice treated with KBrO_3_. Final survival rates of mice treated with 0, 750, or 1500 ppm were 100% (20/20), 90% (18/20), and 75% (15/20), respectively, in *Nrf2* ^+/+^ mice and 84% (21/25), 84% (21/25), and 81% (22/27), respectively, in *Nrf2* ^−/−^ mice.

#### Body weights, water consumptions and chemical intakes

Changes in body weights are illustrated in Figure 1B. Suppression of body weight gains were observed in treated mice of both genotypes from the early exposure period and statistically significant suppression in body weight gains were observed from week 25 until the end of the experiment only in *Nrf2* ^−*/*−^ mice treated with 1500 ppm KBrO_3_. Final body weights of mice treated with 0, 750, or 1500 ppm were 53.5, 51.7, and 49.3 g, respectively, in *Nrf2* ^+/+^ mice and 52.0, 48.5, and 42.9 g, respectively, in *Nrf2* ^−/−^ mice. Average water consumption levels per day for mice receiving 0, 750, and 1500 ppm were 5.6, 4.8, and 4.2 g in *Nrf2* ^+/+^ mice and 5.7, 4.1, and 3.8 g in *Nrf2* ^−*/*−^ mice, respectively. Average chemical intake values per day for mice receiving 750 and 1500 ppm KBrO_3_ were 90.4 and 168.6 mg/kg/mouse, respectively, for *Nrf2* ^+/+^ mice and 81.8 and 163.7 mg/kg/mouse, respectively, for *Nrf2* ^−*/*−^ mice.

#### Histopathological findings

**Figure 2:**
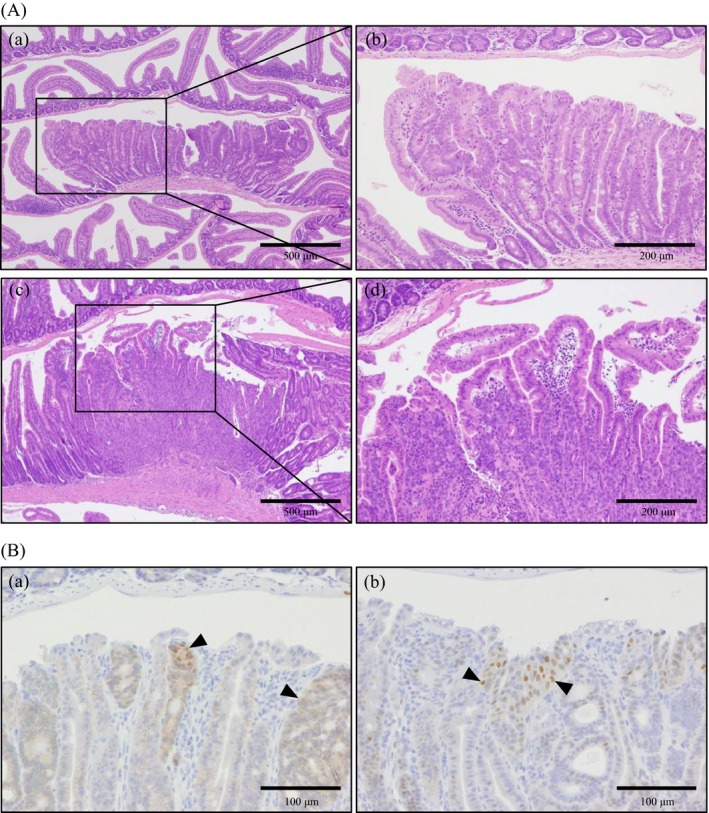
(A) Histopathological features of the small intestines of *Nrf2* ^−*/*−^ mice treated with KBrO_3_ for 52 weeks. (a) Atypical hyperplasia observed in *Nrf2* ^−*/*−^ mice treated with 1500 ppm KBrO_3_. (b) Atypical structures of mucosal epithelia in (a). (c) Adenocarcinoma observed in *Nrf2* ^−*/*−^ mice treated with 1500 ppm KBrO_3_. (d) Infiltration of tumor cells in the lamina propria in mice in (c). Hematoxylin and eosin staining was used. (B) Immunohistochemistry for *β*‐catenin and cyclin D1 in adenoma observed in *Nrf2* ^−*/*−^ mice treated with 1500 ppm KBrO_3_. (a) Increased cytoplasmic and nuclear localizations of *β*‐catenin in atypical epithelial cells (arrow heads). (b) Cyclin D1‐positive signals mainly localized in the nuclei of atypical epithelial cells (arrow heads).

Histopathologically, in *Nrf2* ^−/−^ mice treated with 0, 750 and 1500 ppm, atypical hyperplasia was observed at rates of 5%, 10% and 18%, respectively (Table 1). Moreover, adenoma and adenocarcinoma were observed in each one animal of *Nrf2* ^−*/*−^ mice treated with 1500 ppm, at the rate of 5% in this group, respectively (Table 1). Histopathological features of atypical hyperplasia and adenocarcinoma were shown in Figure 2A. Combined incidences of preneoplastic and neoplastic lesions in *Nrf2* ^−*/*−^ mice treated with 1500 ppm was significantly higher than that in the relative control group and the same dose group in *Nrf2* ^+/+^ mice (Table 1). These neoplastic proliferative lesions were developed predominantly in the upper small intestine (Table 2).

**Table 1:** Incidences and multiplicities of neoplastic proliferative lesions of the small intestine in *Nrf2* ^+/+^ and *Nrf2* ^−/−^ mice treated with KBrO_3_ for 52 weeks

Genotype	*Nrf2* ^+/+^			*Nrf2* ^−/−^		
		KBrO_3_ (ppm)			KBrO_3_ (ppm)	
Dose	Control	750	1500	Control	750	1500
Effective no. of mouse	20	18	16	21	21	22
Preneoplastic lesion						
Atypical hyperplasia	0	0	0	1 (5%)	2 (10%)	4 (18%)
	0	0	0	0.05 ± 0.22	0.19 ± 0.68	0.18 ± 0.39
Neoplastic lesions						
Adenoma	0	0	0	0	0	1 (5%)
	0	0	0	0	0	0.05 ± 0.21
Adenocarcinoma	0	0	0	0	0	1 (5%)
	0	0	0	0	0	0.05 ± 0.21
Combined incidence of preneoplastic and neoplastic lesions	0	0	0	1 (5%)	2 (10%)	6 (27%)^a^^,^ b

a *P *<* *0.05 versus. the relevant control.

b *P *<* *0.05 versus. KBrO_3_ (1500 ppm) in *Nrf2* ^+/+^.

**Table 2:** Site‐specific incidences and multiplicities of neoplastic proliferative lesions in *Nrf2* ^+/+^ and *Nrf2* ^−/−^ mice treated with KBrO_3_ for 52 weeks

Genotype	*Nrf2* ^+/+^			*Nrf2* ^−/−^		
		KBrO_3_ (ppm)			KBrO_3_ (ppm)	
Dose	Control	750	1500	Control	750	1500
Effective no. of mouse	20	18	16	21	21	22
Atypical hyperplasia						
Duodenum	0	0	0	1 (5%)	1 (5%)	0
	0	0	0	0.05 ± 0.22	0.05 ± 0.22	0
Jejunum	0	0	0	0	1 (5%)	4 (18%)
	0	0	0	0	0.05 ± 0.22	0.18 ± 0.39
Ileum	0	0	0	0	1 (5%)	0
	0	0	0	0	0.10 ± 0.44	0
Adenoma						
Duodenum	0	0	0	0	0	0
	0	0	0	0	0	0
Jejunum	0	0	0	0	0	1 (5%)
	0	0	0	0	0	0.05 ± 0.21
Ileum	0	0	0	0	0	0
	0	0	0	0	0	0
Adenocarcinoma						
Duodenum	0	0	0	0	0	0
	0	0	0	0	0	0
Jejunum	0	0	0	0	0	1 (5%)
	0	0	0	0	0	0.05 ± 0.21
Ileum	0	0	0	0	0	0
	0	0	0	0	0	0

#### Immunohistochemistry for *β*‐catenin and cyclin D1

To determine if the neoplastic proliferative lesions observed in *Nrf2* ^−/−^ mice treated with KBrO_3_ have the same characteristics such as accumulation of *β*‐catenin and cyclin D1 as those in *Mutyh* ^−/−^ or *Msh2* ^−/−^ mice given KBrO_3_ 8, 9, immunohistochemistry for *β*‐catenin or cyclin D1 were performed. Because of the size of any lesion being very small, several lesions were unfortunately lost in the re‐slicing paraffin blocks. Therefore, although all of the lesions examined in the immunohistochemistry showed positive, the number of lesions examined was described as follows. Cytoplasm or nuclei of epithelial cells of two atypical hyperplasias observed at 750 ppm and four atypical hyperplasias, one adenoma and one adenocarcinoma observed at 1500 ppm were positive for *β*‐catenin. Nuclei of epithelial cells of one atypical hyperplasia observed at 750 ppm, two atypical hyperplasias and one adenoma observed at 1500 ppm were positive for cyclin D1 (Fig. 2B).

### Short‐term exposure to KBrO_3_

#### Changes in mRNA expression levels of Nrf2‐regulated genes and protein expression level of NQO1

**Figure 3:**
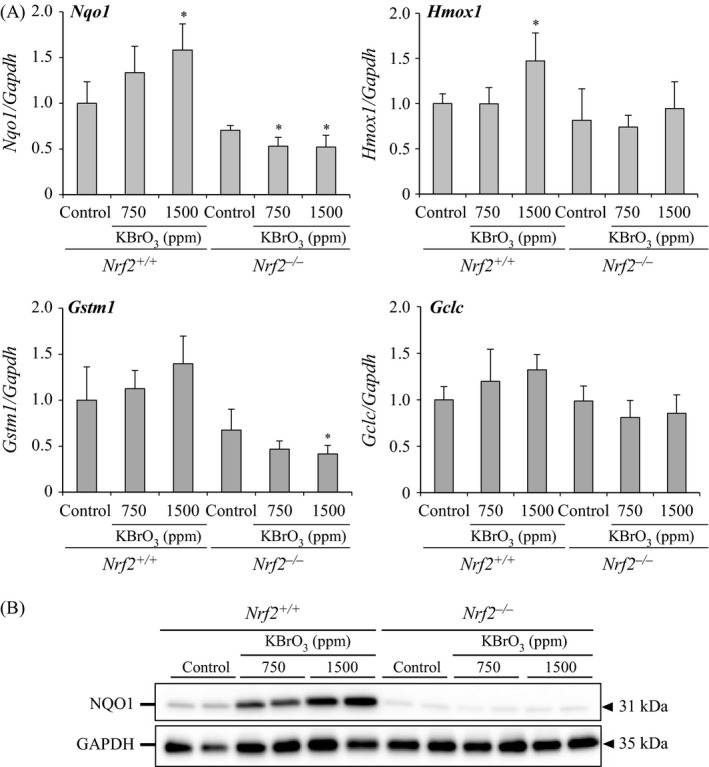
(A) Changes in the expression of Nrf2‐regulated genes (*Nqo1*,* Hmox1*,* Gstm1*, and *Gclc*) in the small intestinal mucosa of *Nrf2* ^+/+^ and *Nrf2* ^−/−^ mice treated with KBrO_3_ for 13 weeks. *Significantly different from relative control group at *P *<* *0.05. (B) Changes in the expression of NQO1 in the small intestinal mucosa of *Nrf2* ^+/+^ and *Nrf2* ^−*/*−^ mice treated with KBrO_3_ for 13 weeks.

Expression levels of Nrf2‐regulated genes were examined. *Nqo1* were dose‐dependently increased in *Nrf2* ^+/+^ mice as compared with that in the control group, with a significant increase at 1500 ppm KBrO_3_. *Hmox1* were significantly increased in *Nrf2* ^+/+^ mice treated with 1500 ppm KBrO_3_. *Gstm1* and *Gclc* were also increased in the same group. In contrast, the expression levels of these genes were not changed or significantly decreased in KBrO_3_‐treated *Nrf2* ^−*/*−^ mice (Fig. 3A). Protein expression level of NQO1 was dose‐dependently increased in KBrO_3_‐treated *Nrf2* ^+/+^ mice, but not changed in *Nrf2* ^−*/*−^ mice (Fig. 3B).

#### Changes in nuclear 8‐OHdG levels

**Figure 4:**
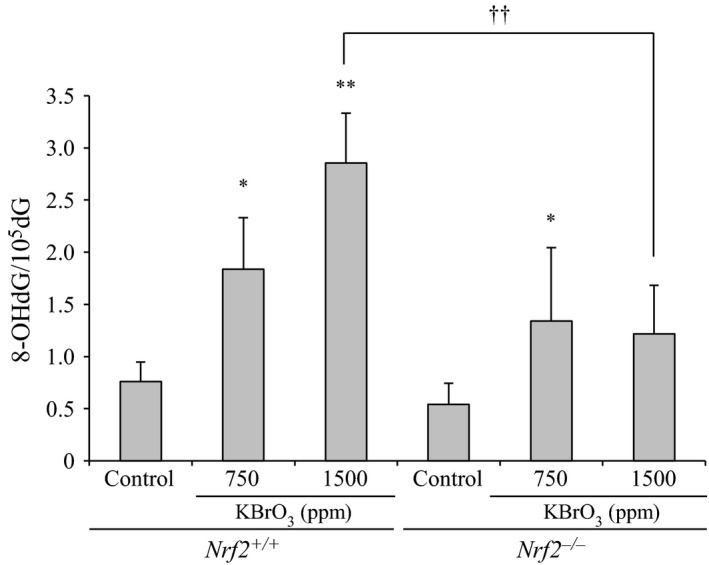
Changes in 8‐OHdG levels in the small intestinal mucosa of *Nrf2* ^+/+^ and *Nrf2* ^−/−^ mice treated with KBrO_3_ for 13 weeks. *,**Significantly different from relative control group at *P *<* *0.05 and 0.01, respectively. ^††^Significantly different from the relative dose group in *Nrf2* ^+/+^ mice at *P *<* *0.01.

The 8‐OHdG levels in the small intestines after 13 weeks of KBrO_3_ administration are shown in Figure 4. Levels of 8‐OHdG were significantly increased in a dose‐dependent manner in KBrO_3_‐treated *Nrf2* ^+/+^ mice compared with that of the control group. In KBrO_3_‐treated *Nrf2* ^−/−^ mice, 8‐OHdG levels were increased at 750 and 1500 ppm as compared with that in the control group, with a significant increase observed only at 750 ppm. 8‐OHdG levels in *Nrf2* ^−/−^ mice treated with 1500 ppm KBrO_3_ were significantly lower than those in *Nrf2* ^+/+^ mice at the same dose.

#### Changes in protein expression levels of COX2 and mRNA expression levels of Il1*β* and Tnf

**Figure 5:**
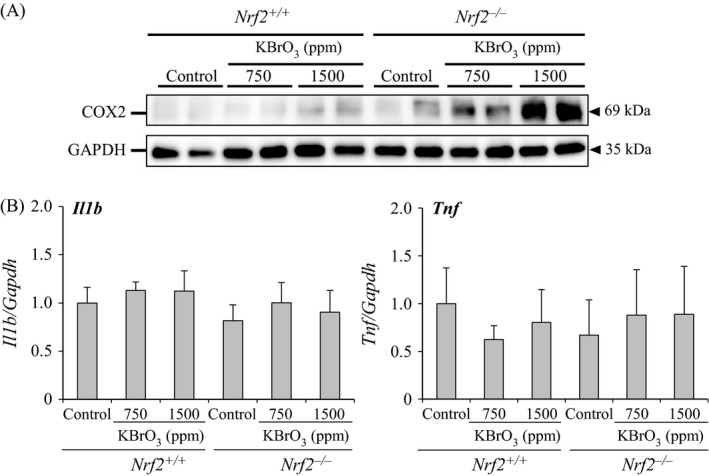
(A) Changes in the protein expression levels of COX2 in the small intestinal mucosa of *Nrf2* ^+/+^ and *Nrf2* ^−/−^ mice treated with KBrO_3_ for 13 weeks. (B) Changes in the expression of genes encoding inflammatory cytokine (i.e., *Il1b* and *Tnf*) in the small intestinal mucosa of *Nrf2* ^+/+^ and *Nrf2* ^−/−^ mice treated with KBrO_3_ for 13 weeks.

To clarify the contributing factor to tumor development in KBrO_3_‐treated *Nrf2* ^−/−^ mice, protein expression levels of COX2 were examined. Since COX2 is induced by inflammation as well as oxidative stress 20, 21, mRNA expression levels of inflammatory cytokines were additionally examined. In *Nrf2* ^+/+^ mice, the protein expression levels of COX2 were slightly increased only at 1500 ppm compared with relative control group. In *Nrf2* ^−/−^ mice, the levels were moderately increased at 750 ppm and severely increased at 1500 ppm compared with relative control group. Protein expression levels of COX2 in *Nrf2* ^−/−^ mice treated with 750 and 1500 ppm KBrO_3_ were higher than those in *Nrf2* ^+/+^ mice with the same treatment (Fig. 5A). The mRNA expression levels of *Il1b* and *Tnf* were not changed in KBrO_3_‐treated mice of both genotypes (Fig. 5B).

#### PCNA analysis of the small intestinal mucosa

**Figure 6:**
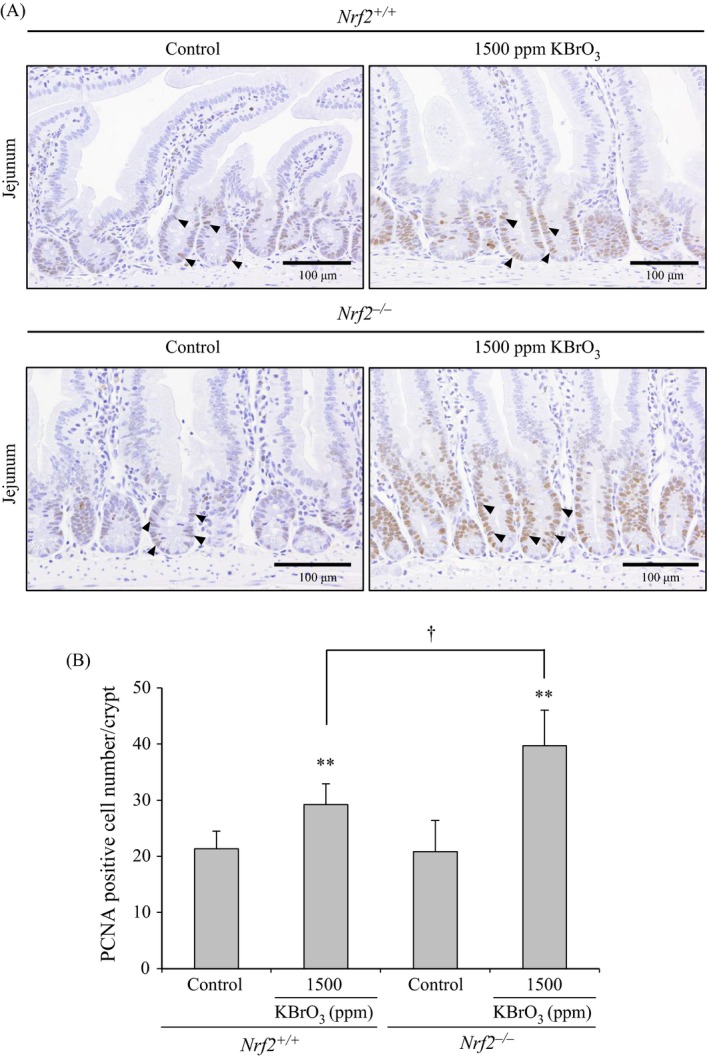
(A) Immunohistochemical staining of PCNA in the jejunum of *Nrf2* ^+/+^ and *Nrf2* ^−*/*−^ mice treated with KBrO_3_ for 13 weeks. Arrow heads show PCNA‐positive nuclei. (B) Numbers of PCNA‐positive cells per crypt. Values are mean ±SD of data for five mice. **Significantly different from the relative control group at *P *<* *0.01. ^†^Significantly different from the relative dose group in *Nrf2* ^+/+^ mice at *P *<* *0.05.

In the jejunum, PCNA‐positive cell numbers per crypt were significantly increased in both of *Nrf2* ^+/+^ mice and *Nrf2* ^−*/*−^ mice treated with 1500 ppm KBrO_3_. PCNA‐positive cell numbers per crypt in *Nrf2* ^−*/*−^ mice treated with 1500 ppm KBrO_3_ were significantly higher than the same dose group in *Nrf2* ^+/+^ mice (Fig. 6A and B). In the duodenum, PCNA‐positive cell numbers per crypt were significantly increased in both of *Nrf2* ^+/+^ mice and *Nrf2* ^−*/*−^ mice treated with 1500 ppm KBrO_3_, with no significant intergenotype changes. In the ileum, PCNA‐positive cell numbers per crypt were not changed in KBrO_3_‐treated mice of both genotypes (data not shown).

## Discussion

In this study, long‐term exposure of *Nrf2* ^−/−^ mice to KBrO_3_ caused atypical hyperplasia, adenoma and adenocarcinoma mainly in the upper part of the small intestine. In most of these lesions, accumulation of *β*‐catenin and cyclin D1 in the nuclei of atypical epithelial cells was observed. These features were consistent with the characteristics of the small intestinal tumors observed in experimental models of human hereditary colorectal cancer using *Mutyh* ^−*/*−^ or *Msh2* ^−*/*−^ mice treated with KBrO_3_ 8, 9, 10. Thus, the neoplastic proliferative lesions observed in this study may have arisen from oxidative stress‐related mechanisms. In fact, increased levels of anti‐oxidant enzymes, *Nqo1*,* Hmox1*,* Gclc,* and *Gstm1* mRNAs and NQO1 protein, were observed in *Nrf2* ^+/+^ mice treated with KBrO_3_ implying that *Nrf2* ^−*/*−^ mice were subjected to increased oxidative stress in the absence of antioxidant enzyme induction.

The incidences of atypical hyperplasia were increased in a dose‐dependent manner in *Nrf2* ^−*/*−^ mice. The combined incidence of preneoplastic and neoplastic lesions in the small intestines of *Nrf2* ^−*/*−^ mice treated with high‐dose KBrO_3_ was significantly higher than that in the control, although adenoma and adenocarcinoma was observed in each one animal in this group, respectively. Because neither preneoplastic nor neoplastic lesions were observed at all doses of KBrO_3_ in *Nrf2* ^+/+^ mice, it is likely that the lack of *Nrf2* was involved in the development of oxidative stress‐associated neoplastic proliferative lesions in the small intestine. However, the incidences and multiplicities of tumors in the small intestine have been reported to be dramatically increased in *Mutyh* ^−*/*−^ or *Msh2* ^−*/*−^ mice treated with KBrO_3_ for 16 weeks 8, 9, 10. KBrO_3_ is able to increase 8‐OHdG in DNA at the carcinogenic target site 22. 8‐OHdG causes G:C‐T:A transversion mutations resulting from its potential for mispairing with adenine 23. Accordingly, lack of repair enzyme for avoiding 8‐OHdG‐related mutagenesis such as Mutyh may result in marked enhancement of KBrO_3_‐induced tumors. Similarly, because Msh2 prevents accumulation of 8‐OHdG and mutations in mouse embryo fibroblasts 6, 7, the same phenomenon may occur in *Msh2* ^−*/*−^ mice. In the present study, exposure of *Nrf2* ^+/+^ mice to KBrO_3_ caused a significant elevation in 8‐OHdG levels in small intestinal DNA in a dose‐dependent manner, whereas exposure of *Nrf2* ^−*/*−^ mice to low‐dose KBrO_3_ caused a significant elevation in 8‐OHdG levels. At the high dose, 8‐OHdG levels in *Nrf2* ^−*/*−^ mice were significantly lower than those in *Nrf2* ^+/+^ mice. However, in view of the small difference in 8‐OHdG levels between the two genotypes, these results are considered not to be sufficient to explain the susceptibility to oxidative stress‐associated intestinal carcinogenesis in *Nrf2* ^−/−^ mice. KBrO_3_ is a potent oxidizing agent and is capable of inducing oxidative stress to generate not only oxidized DNA bases but also other oxidized products such as products of lipid peroxidation 24. Indeed, in this study, KBrO_3_ caused activation of the Nrf2 pathways in *Nrf2* ^+/+^ mice, as described above. Therefore, the dramatic difference in the tumor incidence in the small intestine following KBrO_3_ exposure in *Nrf2* ^−/−^ and *Mutyh* ^−/−^ mice may be responsible for the differences in sites of action against KBrO_3_‐induced oxidative stress.

Considered from a different viewpoint on these facts, pathways other than 8‐OHdG formation may lead to tumor development through oxidative stress in KBrO_3_‐treated *Nrf2* ^−/−^ mice. In the present study, short‐term exposure of *Nrf2* ^−*/*−^ mice to KBrO_3_ markedly increased COX2 protein levels in a dose‐dependent manner compared with that in *Nrf2* ^+/+^ mice. COX2 is induced by various factors, such as inflammation and oxidative stress 20, 21. Since there were no increases in expression levels of mRNA encoding inflammatory cytokines such as *Il1b* and *Tnf*, oxidative stress induced by KBrO_3_ may be a trigger for COX2 induction. Moreover, it is well known that increased COX2 expression results in cell cycle progression 21. This study provided evidence showing that the PCNA‐positive cell numbers in jejunal crypts were increased. Thus, oxidative stress induced by KBrO_3_ failed to fully oxidize DNA bases, but was able to induce COX2 overexpression, leading to cell cycle progression. COX2 has been shown to be involved in promotion of intestinal polyp formation in *Apc* ^min/+^ mice, a model of familial adenomatous polyposis (FAP) 25, 26. Therefore, overexpression of COX2 and subsequent stimulation of cell cycle progression may contribute to the development of neoplastic proliferative lesions in the small intestines of *Nrf2* ^−*/*−^ mice treated with KBrO_3_. In humans, overexpression of COX2 is associated with colorectal carcinogenesis. Specifically, there is a close relationship between the extent of COX2 expression and the size of human colonic adenoma 27. Moreover, overexpression of COX2 is found in subepithelial interstitial cells of human colonic adenoma 28, and the COX2‐inhibitor celecoxib significantly reduces the relapse of adenoma in the colon after polypectomy 29. Thus, *Nrf2* ^−*/*−^ mice are subjected to increased oxidative stress induced by KBrO_3_, and consequent COX2 overexpression would induce cell cycle progression, thereby contributing to the development of neoplastic proliferative lesions.

In conclusion, the present data showed for the first time that *Nrf2* deficiency increased susceptibility to oxidative stress‐induced small intestinal carcinogenesis in mice; this mechanism involved overexpression of COX2 due to oxidative stress, followed by stimulation of cell cycle progression. Overall, our data suggested that individuals with SNPs in *NRF2* may have risk of colorectal cancer to some extent.

## Conflict of Interest

The authors have no conflicts of interest. However, Y. Y. is an employee of Ono Pharmaceutical Co. Ltd.

## References

[1] van der Logt, E. M. , H. M. Roelofs , T. Wobbes , F. M. Nagengast , and W. H. Peters . 2015 High oxygen radical production in patients with sporadic colorectal cancer. Free Radic. Biol. Med. 39:182–187. 15964509 10.1016/j.freeradbiomed.2005.03.003

[2] Łukasz, M. , S. Andrzej , T. Radzisław , G. Krzysztof , R. Maciej , and D. Adam . 2010 Oxidative protein damage in patients with colorectal cancer. Pol. J. Surg. 82:454–458.

[3] Yamaguchi, S. , H. Ogata , D. Katsumata , M. Nakajima , T. Fujii , S. Tsutsumi , et al. 2014 MUTYH‐associated colorectal cancer and adenomatous polyposis. Surg. Today 44:593–600. 23605219 10.1007/s00595-013-0592-7

[4] Silva, F. C. , M. D. Valentin , O. Fde Ferreira , D. M. Carraro , and B. M. Rossi . 2009 Mismatch repair genes in Lynch syndrome: a review. Sao Paulo Med J 127:46–51. 19466295 10.1590/S1516-31802009000100010 PMC10969316

[5] Pistorius, S. , H. Görgens , J. Plaschke , R. Hoehl , S. Krüger , C. Engel , et al. 2007 Genomic rearrangements in MSH2, MLH1 or MSH6 are rare in HNPCC patients carrying point mutations. Cancer Lett. 248:89–95. 16837128 10.1016/j.canlet.2006.06.002

[6] Colussi, C. , E. Parlanti , P. Degan , G. Aquilina , D. Barnes , P. Macpherson , et al. 2002 The mammalian mismatch repair pathway removes DNA 8‐oxodGMP incorporated from the oxidized dNTP pool. Curr. Biol. 12:912–918. 12062055 10.1016/s0960-9822(02)00863-1

[7] Russo, M. T. , G. De Luca , I. Casorelli , P. Degan , S. Molatore , F. Barone , et al. 2009 Role of MUTYH and MSH2 in the control of oxidative DNA damage, genetic instability, and tumorigenesis. Cancer Res. 69:4372–4379. 19435918 10.1158/0008-5472.CAN-08-3292

[8] Isoda, T. , Y. Nakatsu , K. Yamauchi , J. Piao , T. Yao , H. Honda , et al. 2014 Abnormality in Wnt signaling is causatively associated with oxidative stress‐induced intestinal tumorigenesis in MUTYH‐null mice. Int. J. Biol. Sci. 10:940–947. 25170306 10.7150/ijbs.9241 PMC4147226

[9] Piao, J. , Y. Nakatsu , M. Ohno , K. Taguchi , and T. Tsuzuki . 2013 Mismatch repair deficient mice show susceptibility to oxidative stress‐induced intestinal carcinogenesis. Int. J. Biol. Sci. 10:73–79. 24391453 10.7150/ijbs.5750 PMC3879593

[10] Sakamoto, K. , Y. Tominaga , K. Yamauchi , Y. Nakatsu , K. Sakumi , K. Yoshiyama , et al. 2007 MUTYH‐null mice are susceptible to spontaneous and oxidative stress induced intestinal tumorigenesis. Cancer Res. 67:6599–6604. 17638869 10.1158/0008-5472.CAN-06-4802

[11] Keum, Y. S. , and B. Y. Choi . 2014 Molecular and chemical regulation of the Keap1‐Nrf2 signaling pathway. Molecules 19:10074–10089. 25014534 10.3390/molecules190710074 PMC6270911

[12] Marzec, J. M. , J. D. Christie , S. P. Reddy , A. E. Jedlicka , H. Vuong , P. N. Lanken , et al. 2007 Functional polymorphisms in the transcription factor NRF2 in humans increase the risk of acute lung injury. FASEB J. 21:2237–2246. 17384144 10.1096/fj.06-7759com

[13] Arisawa, T. , T. Tahara , T. Shibata , M. Nagasaka , M. Nakamura , Y. Kamiya , et al. 2007 The relationship between Helicobacter pylori infection and promoter polymorphism of the Nrf2 gene in chronic gastritis. Int. J. Mol. Med. 19:143–148. 17143558

[14] Arisawa, T. , T. Tahara , T. Shibata , M. Nagasaka , M. Nakamura , Y. Kamiya , et al. 2008 Nrf2 gene promoter polymorphism is associated with ulcerative colitis in a Japanese population. Hepatogastroenterology 55:394–397. 18613373

[15] Itoh, K. , T. Chiba , S. Takahashi , T. Ishii , K. Igarashi , and Y. Katoh , et al. 1997 An Nrf2/small Maf heterodimer mediates the induction of phase II detoxifying enzyme genes through antioxidant response elements. Biochem. Biophys. Res. Commun. 236:313–322. 9240432 10.1006/bbrc.1997.6943

[16] Kurokawa, Y. , A. Maekawa , M. Takahashi , and Y. Hayashi . 1990 Toxicity and carcinogenicity of potassium bromate–a new renal carcinogen. Environ. Health Perspect. 87:309–335. 2269236 10.1289/ehp.9087309 PMC1567851

[17] Betton, G. R. , L. O. Whiteley , M. R. Anver , R. Brown , U. Deschl , M. Elwell , et al. 2001 Gastrointestinal tract Pp. 23–58 *in* Mohr U., ed. International classification of rodent tumors: the mouse. Springer‐Verlag, Berlin, Germany.

[18] Umemura, T. , S. Kai , R. Hasegawa , K. Kanki , Y. Kitamura , A. Nishikawa , et al. 2003 Prevention of dual promoting effects of pentachlorophenol, an environmental pollutant, on diethylnitrosamine‐induced hepato‐ and cholangiocarcinogenesis in mice by green tea infusion. Carcinogenesis 24:1105–1109. 12807750 10.1093/carcin/bgg053

[19] Umemura, T. , K. Kanki , Y. Kuroiwa , Y. Ishii , K. Okano , T. Nohmi , et al. 2006 In vivo mutagenicity and initiation following oxidative DNA lesion in the kidneys of rats given potassium bromate. Cancer Sci. 97:829–835. 16805826 10.1111/j.1349-7006.2006.00248.x PMC11158994

[20] Sun, Y. , J. Chen , and B. Rigas . 2009 Chemopreventive agents induce oxidative stress in cancer cells leading to COX‐2 overexpression and COX‐2‐independent cell death. Carcinogenesis 30:93–100. 18952595 10.1093/carcin/bgn242 PMC2639032

[21] Konturek, P. C. , J. Kania , G. Burnat , E. G. Hahn , and S. J. Konturek . 2005 Prostaglandins as mediators of COX‐2 derived carcinogenesis in gastrointestinal tract. J. Physiol. Pharmacol. 56(Suppl 5):57–73. 16247189

[22] Umemura, T. , M. Tasaki , A. Kijima , T. Okamura , T. Inoue , Y. Ishii , et al. 2009 Possible participation of oxidative stress in causation of cell proliferation and in vivo mutagenicity in kidneys of *gpt* delta rats treated with potassium bromate. Toxicology 257:46–52. 19133310 10.1016/j.tox.2008.12.007

[23] Cheng, K. C. , D. S. Cahill , H. Kasai , S. Nishimura , and L. A. Loeb . 1992 8‐Hydroxyguanine, an abundant form of oxidative DNA damage, causes G‐T and A‐C substitutions. J. Biol. Chem. 267:166–172. 1730583

[24] Umemura, T. , Y. Kitamura , K. Kanki , S. Maruyama , K. Okazaki , T. Imazawa , et al. 2004 Dose‐related changes of oxidative stress and cell proliferation in kidneys of male and female F344 rats exposed to potassium bromate. Cancer Sci. 95:393–398. 15132765 10.1111/j.1349-7006.2004.tb03221.x PMC11158485

[25] Cherukuri, D. P. , T. O. Ishikawa , P. Chun , A. Catapang , D. Elashoff , T. R. Grogan , et al. 2014 Targeted Cox2 gene deletion in intestinal epithelial cells decreases tumorigenesis in female, but not male, *Apc* ^Min/+^ mice. Mol. Oncol. 8:169–177. 24268915 10.1016/j.molonc.2013.10.009 PMC3963510

[26] Cheung, K. L. , J. H. Lee , T. O. Khor , T. Y. Wu , G. X. Li , J. Chan , et al. 2014 Nrf2 knockout enhances intestinal tumorigenesis in Apc(min/+) mice due to attenuation of anti‐oxidative stress pathway while potentiates inflammation. Mol. Carcinog. 53:77–84. 22911891 10.1002/mc.21950

[27] Pisano, C. , A. Ottaiano , F. Tatangelo , M. Di Bonito , M. Falanga , V. R. Laffaioli , et al. 2005 Cyclooxygenase‐2 expression is associated with increased size in human sporadic colorectal adenomas. Anticancer Res. 25:2065–2068. 16158946

[28] Chapple, K. S. , E. J. Cartwright , G. Hawcroft , A. Tisbury , C. Bonifer , N. Scott , et al. 2000 Localization of cyclooxygenase‐2 in human sporadic colorectal adenomas. Am. J. Pathol. 156:545–553. 10666384 10.1016/S0002-9440(10)64759-1 PMC1850032

[29] Arber, N. , C. J. Eagle , J. Spicak , I. Rácz , P. Dite , J. Hajer , et al. 2006 Celecoxib for the prevention of colorectal adenomatous polyps. N. Engl. J. Med. 355:885–895. 16943401 10.1056/NEJMoa061652

